# Addition to Staff

**Published:** 1913-03

**Authors:** 


**Addition to Staff**

Dr. J. George Adami of Montreal, who joins the Foreign Staff of the JOURNAL, is too well known by his work in pathology and has too many personal friends among us, to require an introduction. We extend to him a cordial welcome. The delay in having Canada represented on our staff has been due to the fact that it was only recently that it occurred to us that Canada was a foreign country. It is said that there are more Yankees in proportion to the population in Ontario, between the two lakes, than in any part of the United States, not to mention the fact that the earlier settlers were of essentially the same blood as the Yankees.

